# Genetic Evidence Confirms Polygamous Mating System in a Crustacean Parasite with Multiple Hosts

**DOI:** 10.1371/journal.pone.0090680

**Published:** 2014-03-07

**Authors:** Quentin Jossart, Rémi A. Wattier, Chedly Kastally, Serge Aron, Bruno David, Chantal De Ridder, Thierry Rigaud

**Affiliations:** 1 Département de Biologie des Organismes, Laboratoire de Biologie Marine, Université Libre de Bruxelles (ULB), Brussels, Belgium; 2 Biogéosciences (UMR CNRS 6282), Université de Bourgogne, Dijon, France; 3 Département de Biologie des Organismes, Behavioral and Evolutionary Ecology, Université Libre de Bruxelles (ULB), Brussels, Belgium; Columbia University, United States of America

## Abstract

Mating systems are diverse in animals, notably in crustaceans, but can be inferred from a limited set of parameters. Baeza and Thiel (2007) proposed a model predicting mating systems of symbiotic crustaceans with three host characteristics and the risk of predation. These authors proposed five mating systems, ranging from monogamy to polygynandry (where multiple mating occurs for both genders). Using microsatellite loci, we tested the putatively mating system of the ectoparasite crab *Dissodactylus primitivus*. We determined the mating frequencies of males and females, parentage assignment (COLONY & GERUD software) as well as the contents of female spermathecae. Our results are globally consistent with the model of Baeza and Thiel and showed, together with previous aquarium experiments, that this ectoparasite evolved a polygamous mating system where males and females move between hosts for mate search. Parentage analyses revealed that polyandry is frequent and concerns more than 60% of clutches, with clutches being fertilized by up to 6 different fathers. Polygyny is supported by the detection of eight males having sired two different broods. We also detected a significant paternity skew in 92% of the multipaternal broods. Moreover, this skew is probably higher than the estimation from the brood because additional alleles were detected in most of spermathecae. This high skew could be explained by several factors as sperm competition or cryptic female choice. Our genetic data, combined with previous anatomic analyses, provide consistent arguments to suggest sperm precedence in *D. primitivus*.

## Introduction

The knowledge of mating systems is of primary importance to determine the factors that have shaped evolutionary trends in a given taxon. In return, phylogenetic or ecological constraints may also explain why a mating system could be predominant in a biological group. Mating systems are diverse in animals, but can be predicted using a limited set of parameters that determine the intensity of sexual selection, such as anisogamy, operational sex ratio and the spatial and temporal distribution of ready-to-mate individuals (especially receptive females) [1], [2], [3].

Crustaceans are extraordinarily diverse in morphology, life history traits and habitat distribution [3], [4], [5]. They are ideal biological models for the study of the selective forces affecting the evolution of mating systems, because closely related species can evolve different lifestyles and mating strategies. This prospect is reinforced by the variability of many traits linked with sexual selection, including internal vs. external fertilization, presence vs. absence of sperm storage, intensive vs. no parental care, semelparity vs. iteroparity [6], [7]. Surprisingly, however, their mating systems remain largely unexplored, with the noticeable exception of commercially - exploited species [8], [9], [10].

Several crustacean taxa, including isopods, amphipods, shrimps and crabs have evolved a symbiotic life history strategy. Symbiotic crustaceans live with various other invertebrates (sponges, anthozoans, sea urchins, among others), with relationships ranging from parasitism to commensalism [4]. For these crustacean symbionts, the hosts are discrete habitats providing food, shelter and mating site. Such a discrete distribution of breeding habitats may deeply influence mating systems, because the distribution and abundance of host affect symbiont behavior and, hence, the rate and number of interactions between potential mates. Based on the framework of Shuster & Wade [2] and on life history strategies, Baeza and Thiel [3] proposed a model predicting mating systems in symbiotic crustaceans as a function of three host characteristics, namely the relative size of host vs. symbiont, host morphological complexity, and host abundance, as well on the predation risk off hosts [5]. According to their model, these traits affect directly the tendency of symbiotic crustaceans to either monopolize their hosts (host guarding behavior, if hosts are not too large, rare and if predation risk while changing host is high) or, rather, to roam among them (host switching behavior, if hosts are too large and too complex to be guarded, abundant, and if predation risk off hosts is low). Monopolizing hosts or changing between them are the extremes of a continuum, where the rate and number of interactions between individuals directly influence mating opportunities. Baeza and Thiel's model comprises five mating systems, ranging from strict monogamy to polygynandry (i.e., mating occurs between multiple males and females with higher variance in mate numbers for males). Surprisingly, polyandrogyny, which is characterized by a higher variance in mate numbers for females, is not considered in this theoretical model [2]. Interestingly, because the model focuses on mating systems in discontinuous habitat, it could be extended to other aquatic or terrestrial organisms living in discrete refuges, like parasitoid insects, litter-associated amphibians or sea-grasses bed associated species. While the authors provided some examples illustrating their model, they also claimed that empirical studies are lacking to confirm its ubiquity [3], [11]. Over the last decade, a number of works explored the mating systems of ectosymbiotic crustaceans to test Baeza and Thiel's model. Most relied, on observations of life-cycles or of life histories [3], [5], [11], [12], [13], [14], [15], [16]. However, due to the aquatic lifestyle of ectosymbiotic crustaceans, direct observations are not easy and the knowledge on the species' mating system is often incomplete. Though polygamy or monogamy was suspected in a number of cases, there was a lack of direct evidence [11], [12], [15]. More recently, mating systems have been investigated using molecular tools, especially microsatellite loci [8], [9], [17], [18]. These studies showed that multiple mating occurs rather frequently in crustaceans [18], [19] but none of them concerns ectosymbiotic species.

In the present work, we used the ectosymbiotic crab *Dissodactylus primitivus* to test the prediction of the model of Baeza and Thiel [3] that mating system can be inferred from host and crustacean life history traits. *D. primitivus* is a pea-crab (Pinnotheridae), ectoparasite of two burrowing sea urchins (*Meoma ventricosa* and *Plagiobrissus grandis*) distributed along the Caribbean and neighboring American coasts [11], [20], [21]. A higher fertility in pea-crab females living on *P. grandis* was observed [13]. A population genetics analysis revealed no genetic differentiation among crabs living on the two different host species, suggesting a lack of host specialization [22]. Consequently, the higher fertility of females living on *P. grandis* remained unexplained. Considering Baeza and Thiel's model, the authors hypothesized that *D. primitivus* could use a strategy of “pure-search polygynandry of mobile females” whereby both males and females move between hosts to find several mates at the period of reproduction. Indeed, individual hosts are relatively close to each other with population densities of 0.2 individuals/m^2^ for *M. ventricosa* and 0.02 individuals/m^2^ for *P. grandis*. Host sizes are also particularly large compared to their symbiotic crabs (*ca*. 225 times larger in area). Finally, predation risk undergone by the crabs when changing hosts could be low because they display the same color as the surrounding coral sands. Using demographic analyses, De Bruyn et al. [11], [13] showed that *D. primitivus* assemblages living on a same host are variable in composition. Crabs can be found alone, in pairs (heterosexual or homosexual), and up to 6 adult crabs can infect a single host [11,unpublished data]. Furthermore, both genders could move among host individuals, whatever the host species [11], [13]. If a “pure-search polygynandry of mobile females” mating system actually occurs, a high rate of polyandry and polygyny should be genetically detected.

Using microsatellite marker loci, we tested the “pure-search polygynandry of mobile females” hypothesis in *D. primitivus*. We determined the mating frequencies of males and females, as well as parentage assignment by genotyping eggs from gravid females and the contents of female spermathecae. In addition we addressed the following two questions: (i) does mating system of *D. primitivus* vary between the two hosts? This could account for the difference in female fertility if mating frequency is positively associated with fitness on *P. grandis* or, conversely, if females suffer sexual harassment on *M. ventricosa*; and (ii) is multiple mating associated to an unequal contribution of the females' mates to offspring production.

## Materials and Methods

### Sampling

Crabs were sampled in one site, located into the lagoon of Discovery Bay (180°28′N, 77°24′W, Northern coast of Jamaica) in March-April 2009 by SCUBA diving or snorkeling at depths ranging from 2 to 4 m. All samples were obtained under the University of the West Indies Collecting Permit from the National & Environmental Planning Agency.

Fifteen hosts were sampled separately in plastic bags that were immediately tied up after collection. Back in the laboratory, crabs were individually isolated and preserved in pure ethanol. We collected 39 gravid crab females (for a total of 64 females), among which 18 were chosen for molecular analyses (9 from 8 *M. ventricosa* and 9 from 7 *P. grandis*) (Table 1). All collected male crabs at the same site (55 in total) were used for molecular analyses, taking in account their host of origin (labeled “M” or “P” crabs sampled on *M. ventricosa* or *P. grandis*, respectively). Gravid females carried 203 eggs±34 (mean ± SD, N = 9). Genotyping was performed on an average of 40.56±4.67 eggs per clutch, totaling 758 eggs that were randomly sampled in each clutch (Table 1). For 14 of these 18 gravid females, we also collected a spermatheca.

**Table 1:** Characteristics of clutches, host infrapopulation composition and estimated fathers number and identity.

Mother's (clutch) ID (a)	No. of eggs genotyped	No. of fathers (b)	Adult infrapop. (c)		Fatherhood (d)		
				
			f	m	m1	m2	m3
M12	46	2/2	2(1)	1	M15		1
M16	44	1/2	1(1)	1			**2** ^*^ 3
M22	31	1/1	2(1)	1	M24		
M32	39	1/1	2(1)	1	M31		
M39	47	1/1	1(1)	1	M38		
M49	37	2/2	2(1)	0			**4** ^*^ 5
M64	36	4/6	1(1)	1	M63	M18 M84^*^	6^*^ 7^*^ 8^*^
M69	35	2/3	2(2)	1		M84^*^	2^*^ 7^*^
M70	45	3/3	2(2)	1	M71		9 10
P3	42	2/2	1(1)	1			6^*^ 8^*^
P5	45	2/3	1(1)	0			11 12 13^*^
P11	41	2/2	1(1)	2			14 15
P26	38	1/1	1(1)	3	P27		
P46	37	1/1	1(1)	1	P45		
P58	43	1/1	5(2)	3			16^*^
P59	35	2/2	5(2)	3			16^*^ 17
P69	45	3/4	3(2)	0			13^*^ 18 19 20
P70	44	3/3	3(2)	0			4^*^ 21 22
Mean (SD)	40.56 (4.67)	1.89 (0.90)/ 2.22 (1.31)					

(a) M  =  *M. ventricosa* and P  =  *P. grandis*, M69&M70, P58&P59, P69&P70 were found on the same host individual; (b) No. of fathers determined by GERUD/COLONY; (c) composition of the adult infrapopulation (individuals on the same host individual) including the studied mother, f  =  females (including gravid females in brackets) and m  =  males; (d) Fatherhood (COLONY analyses), Males were subdivided in three classes: m1  =  fathers present with the mother on the host, m2  =  males sampled in Discovery Bay and m3  =  inferred fathers, not sampled. Males in bold and shown by a star were contributing to two clutches.

### Tissue and DNA extractions

For each adult crab, two legs were removed and dried during two hours at ambient temperature. The clutch of each gravid female was isolated and eggs were placed individually in a microtube and dried during two hours. Then, the legs and eggs were frozen at −80°C pending DNA extraction step.

DNA extractions were performed using a Chelex chelating resin method [23]. Each sample was crushed using one tungsten ball (3 mm diameter) and a mixer mill (1 min at 18 Hz). After having added 100 µl of “Chelex solution” (1 g of Chelex into 20 ml of sterile water), another crushing was made during 1 min. The samples were then placed at 85°C during 90 min and mixed every 30 min. Finally, after centrifugation (3 min at 12 000 rpm), the supernatant with DNA was collected.

Spermathecae were dried for one hour and then directly placed into Chelex solution without any crushing step.

### Amplification and genotyping

Four highly informative microsatellite loci [24] were amplified in one multiplex (DpA113-VIC, DpA101-NED, DpD110-PET, DpD111-FAM) (Table 2). PCR reactions were made in a volume of 15 µl that includes 7.5 µl of Master Mix Qiagen (Taq Polymerase, nucleotides), 1 µl of DNA, 0.3 µl (10 µM) of each forward/reverse primer and 4.1 µl of sterile water. The PCR conditions consisted of 40 cycles of 30 s at 94°C (denaturation), of 90 s at 51°C (annealing) and of 30 s at 72°C (elongation). These cycles were preceded by a step of 15 min at 95°C (first denaturation) and were followed by a step of 10 min at 72°C (last elongation). Finally, 1 µl of amplified DNA was mixed with 0.4 µl of the size standard LIZ (AB) and 10 µl of formamide prior to electrophoresis with an AB 3730 DNA Analyzer.

**Table 2:** The four microsatellite loci used in this study.

Locus	Motif	Primers	Fluorochrome	Size range (bp)	A
DpA113	AC	F: GCGTAGTTCTCCTCCCGTAG	VIC	108–134	12
		R: GCGCTACCCATCAGTCTTG			
DpA101	CA	F: CTCTCCGTCACTTTGTGTAGGT	NED	217–259	16
		R: GTGTTCTTGTGTGCGTGTATTC			
DpD110	TAGA	F: GAAGGGTTGCTTATAGACGTG	PET	236–276	17
		R: CCTCCTTGTTTACCGTGAGT			
DpD111	CTAC	F: CTTGACCTGACCTGTCTATCA	FAM	251–309	11
		R: CGGTGGACTACATAAGTAAAGG			

A denotes the total number of alleles evaluated from a previous population genetics study [22]. The fluorochromes are part of the DS33 Applied Biosystems Standard Dye Set.

Genotypes were deduced from electropherograms using the software Peak Scanner (products.appliedbiosystems.com). Allelic binning was done using the program Autobin but each genotype was checked by eye [25].

### Parentage analyses

Using Fstat [26], we assessed deviation from Hardy-Weinberg equilibrium using *F_IS_* and randomization of alleles between individuals for 169 previously genotyped specimens, 82 and 87 individuals from *P. grandis* and *M. ventricosa*, respectively [22]. Then, we tested if the four loci were adequate for parentage analysis. First, using the GERUD (2.0) software, we calculated an exclusion probability (PE), namely, the probability to exclude a candidate parent if this candidate is effectively unrelated to the offspring [27], [28]. This PE was estimated taking into account that one parent is known with certainty (here the mother) and using the allelic frequencies based on all adult individuals collected in Discovery Bay (this study and [22]). We also calculated the probability to detect multiple paternity in a sample of the offspring (PrDM) using the software PrDM [29]. This software allows calculation of a set of PrDM according to the number of putative fathers, their relative contribution to offspring and the number of offspring analysed by clutch [9], [29]. For example, given the level of informativeness of our set of loci, we may have increased the number of offspring in order to detect rare contributions. Parentage assignment was analyzed using two software: GERUD 2.0 and COLONY 2.0.2.1 [28], [30], [31]. While GERUD infers the minimum number of fathers, COLONY infers the most likely number of fathers.

Mother's and genotyped offspring's alleles were compared to the set of alleles observed from her spermatheca. Extra alleles were taken as a clue of additional inseminating males for which contribution to clutch was not detected. The number of matings was estimated by summing the number of newly males detected to the minimum number of fathers (GERUD data).

We evaluated how the paternal contributions in each clutch deviated from an equilibrate contribution (G-test for goodness-of-fit on COLONY data, p-value threshold after Bonferroni correction = 0.004) using an Excel Macro developed by McDonald [32]. In addition, skewness (S) in paternity was calculated according to Pamilo and Crozier [33]: S = (M_p_−M_e,p_)/(M_p_-1) where M_p_ is the total number of fathers and M_e,p_ is the effective number of fathers [34]. We also evaluated the correlation between relative contribution of fathers in a clutch and the genetic similarity of the two partners. Genetic similarity (GS) was calculated as GS_ij_ = 2 N_ij_/(N_i_+N_j_) with N_ij_ the number of alleles in common between the partners (i and j) and N_i_ and N_j_ the number of alleles of the individuals i and j, respectively [18], [35].

Other classical statistical tests (Mann-Whitney tests, Spearman correlations, Wilcoxon signed-rank tests, G-tests) were performed using STATISTICA 7.0 (statsoft.com), PAST (folk.uio.no/ohammer/past) or Excel Macro developed by McDonald [32].

## Results

Overall *F_IS_* was equal to −0.025 for adult crabs from *M. ventricosa* and −0.017 for crabs from *P. grandis* and were non-significantly different from zero. This result is in agreement with a previous population genetics study on a larger data set from Discovery Bay [22].

None of the genotypes of the 73 adults used in our study were similar except for two individuals (M16, M17), leading to a probability of exclusion (PE), when one parent is known of 0.991. This indicates that the four microsatellite markers were variable enough to discriminate candidate parents. The probability to detect multiple paternity (PrDM) increased rapidly with sample size, indicating that the selected egg sample size ranging from 35 to 47 was high enough to detect multiple paternity even with unequal contributions of the fathers (Table 3).

**Table 3:** Probabilities to detect multiple paternity (PrDM) for different number of eggs per clutch and either 2 or 3 fathers contributing and different relative contribution.

		Fathers' contributions			
		50∶50	90∶10	33∶33∶33	80∶10∶10
		2 fathers		3 fathers	
**Number of eggs genotyped per clutch**	**10**	0.995	0.635	0.999	0.882
	**20**	0.999	0.867	0.999	0.986
	**30**	0.999	0.950	0.999	0.998
	**40**	0.999	0.981	0.999	0.999
	**50**	0.999	0.992	0.999	0.999

(e.g. 50∶50 is an equal contribution of both fathers).

### Clutch analysis: searching for evidence of polyandry and polygyny in *D. primitivus*

Multiple paternity was detected in 12 clutches out of 18 in COLONY (i.e., 66.7%) and 11 clutches in GERUD (i.e., 61.1%; Table 1). Using GERUD, the minimal mean number of fathers per clutch was 1.89 when combining data from both hosts (Table 1, *M. ventricosa* SD = 1.05 and range = 1–4 and *P. grandis* SD = 0.78, range = 1–3). Using COLONY, the estimated number of fathers per clutch was 2.22 for the whole data set (Table 1), 2.33 for females from *M. ventricosa* (SD = 1.58, range = 1–6) and 2.11 for females from *P. grandis* (SD = 1.05, range = 1–4). These values (between software) were non-significantly different (Mann-Whitney test, U = 142.5, p = 0.54). Unless specified, most of the results described in the forthcoming paragraphs will refer to results obtained with COLONY, because the use of sampled fathers in parentage assignment was possible with this software.

The estimated rate of polyandry in *D. primitivus* was the same on both host species (6/9), and the number of fathers per clutch was not significantly different between the two hosts (Mann-Whitney test, U = 40.0, p = 0.96). The total number of fathers for the whole data set was 32 (Table 1). Among them, COLONY detected eight males that contributed to two different clutches (Table 1), one being among our sampled males (M84) and seven inferred by COLONY. Three fathers contributed to two clutches sampled from the two different host species (males 4; 6 and 8 on Table 1). Three pairs of females (M69–M70, P58–P59, P69–P70) were collected on the same host individual (Table 1). Two of these pairs were sired by different males, but the two females P58–P59 were sired by the same male (male 13, not captured in our sampling).

**Figure 1:**
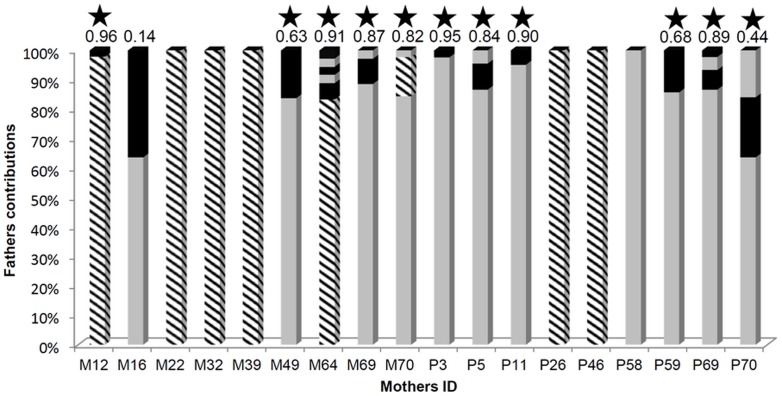
Fathers' contributions within each clutch (COLONY analyses). Fathers are shown by alternate shadings. Stripped bars correspond to fathers sampled on the same host individual as the mother (see also Table 1). The values above bars correspond to the skewness (S) in paternity. The stars indicate that paternal contributions deviated significantly from equality (G-test for goodness-of-fit, Bonferroni adjusted p-value threshold = 0.004). M  =  *M. ventricosa* and P  =  *P. grandis*.

There was a significant deviation from an equal contribution among fathers in 11 out of 12 multipaternal clutches (Figure 1, Table S1), with a skewness of paternity (S) ranging from 0.44 (P70) to 0.96 (M12) with an average of 0.81 (SD = 0.16) (G-tests all significant, all p<0.004). For 8 clutches out of 18, one father was found on the same individual host than the mother (M15, M24, M31, M38, M63, M71, P27, P45; Table 1; Figure 1). Among these 8 fathers, 7 were the principal (or the only one) contributors of the clutch (Figure 1). The mean number of fecundated clutches by a successful male is 1.25 (SD = 0.43, N = 32). The mean number of offspring produced were 114.19 (SD = 98.37, N = 32) for males and 203 (SD = 34, N = 9) for females (Table S2).

There was no association between the number of eggs per clutch (a correction for female size was included by dividing the number of eggs per clutch by the square of the cephalothorax width) and the mating frequency (Spearman correlation, r_s_ = 0.39, p = 0.30, N = 9). Moreover, no correlation was detected between genetic similarity of the two parents and the relative contribution of fathers to the clutch (r_s_ = 0.02, p = 0.90, N = 40).

### Spermatheca analysis: searching for mates that did not contribute to the clutch

The total number of additional alleles detected was different between loci (DpA113: 4, DpA101: 12, DpD111: 23, DpD110: 7). For the 14 spermathecae investigated, nine contained at least a non-parental allele for two loci or more (Table 4). Moreover, two of them had three non-parental alleles for at least two loci (Table 4). There were significantly more mates than the minimum number of fathers (estimated with GERUD) (2.71 *vs* 1.93, Wilcoxon signed-rank test, z = 18.00, p = 0.008).

**Table 4:** Number of non-parental alleles detected in each female's spermatheca for the four loci (DpA113, DpA101, DpD111, DpD110).

Mother's ID	DpA113	DpA101	DpD111	DpD110	Min. no. of fathers	Min. no. of matings
M12	0	1	2	0	2	3
M22	0	0	1	1	1	2
M32	1	0	0	0	1	1
M39	0	1	2	0	1	2
M49	0	1	2	0	2	3
M64	0	0	0	0	4	4
M69	0	0	0	0	2	2
M70	0	0	2	0	3	3
P5	0	1	1	1	2	3
P11	2	2	4	3	2	4
P26	0	0	1	0	1	1
P58	1	2	3	2	1	2
P59	0	3	4	0	2	4
P69	0	1	1	0	3	4
Mean (SD)					1.93 (0.92)	2.71 (1.07)

The minimum number of matings was calculated in the same way than the Minimum number of fathers (GERUD data) to which we added the number of new males deduced from spermatheca analysis.

## Discussion

Our results are globally consistent with the model of Baeza and Thiel [3], indicating that mating system can be predicted from host and crustacean life history traits. They show, together with the experiments of De Bruyn et al. [11], [13], that the ectoparasite crab *D. primitivus* evolved a polygamous mating system where both males and females move between hosts for mate search. Other studies inferred mating systems of symbiotic crustaceans from this predictive model [12], [14], [16]. However, our study is the first that directly links such predictions with genetic measurements. Parentage analyses reveal that polyandry is frequent in *Dissodactylus primitivus* and concerns more than 60% of clutches, with clutches being fertilized by up to 6 different fathers. Moreover, the number of matings was greater than the number of fathers, indicating that some male mates did not contribute to the offspring. Because we did not genotyped the whole clutches, the difference between the minimum number of fathers and the minimum number of matings could be lower than our estimation. Anyway, our estimation of the minimum number of matings was most likely conservative, because (i) we considered an additional mating only when a new allele was detected at two different loci, and (ii) a competition during amplification step may occur, leading to the non-amplification of some alleles (e.g. short allele dominance [36]), therefore minimizing the number of detected additional alleles. Polygyny is supported by the detection of eight males having sired two different broods. Only twenty five percent of the 32 detected fathers occurred simultaneously with the investigated females on the same individual host. This suggests that the fathers and/or the mothers regularly leave the host after mating. In line with this hypothesis, a few inferred males have mated with females collected on distinct host species, indicating that the crabs could move from one to another host whatever the host species. Overall, these results corroborate those of De Bruyn et al. [11], showing that crabs move between hosts in the field, and Jossart et al. [22] reporting a lack of genetic differentiation between crabs found on the two host species. The model of Baeza & Thiel does not consider the variance in mate numbers between genders. Our results (from clutches) suggest a higher variance in females (mean = 2.22; variance = 1.31; N = 18) than in males (mean = 1.25; variance = 0.44; N = 32). Therefore, according to Shuster & Wade (2003), the mating system of *D. primitivus* could be considered as polyandrogyny rather than polygynandry. However, our study probably underestimates the real values, especially for males, because we lack data on the set of females they mated at a given time.

Our data also indicate that the difference in brood size reported by De Bruyn et al. [11] for *D. primitivus* collected on *P. grandis* and *M. ventricosa* does not imply a difference in multiple paternity: the number of mates is not related to the number of eggs occurring in a brood/clutch, conversely to what was observed in some other species where multiple paternity occurs (e.g. the shrimp *Caridina ensifera* [18]).

We detected a significant paternity skew in 92% of the multipaternal broods. This confirms that high skew could occur in various taxa among decapod crustaceans [37], [38]. The paternal skew is probably higher than the estimates inferred from the brood since additional alleles were detected in most of spermatheca (64%). High skews in sperm usage could be explained by several factors like sperm competition or cryptic female choice [39], [40], [41]. Our study was not designed to test the proximate causes for the sperm use bias. Nevertheless, some explanations can be discussed in the light of our data. First, cryptic female choice can be associated with a negative relationship between reproductive success of sires and their relatedness to mothers, allowing the avoidance of genetic incompatibility or inbreeding [42]. Yet, we found no correlation between genetic similarity of the two parents and sire success, nor an advantage in brood size in polyandrous females. Second, females of *Dissodactylus sp*. could produce more than one clutch during a breeding period or during their entire life [43], [44]. They are able to store sperm, but are also potentially receptive to mating before each new clutch. In pea-crabs, the spermatheca forms a pouch with the oviduct opening at the basis of the vagina where sperm intromission takes place [45]. Consequently, if several spermatophores are inserted successively, the last male sperm would be: (i) in higher numbers than the preceding stored sperm (e.g. due to sperm mortality), (ii) in favorable position, at the basis of the spermatheca close to the oviduct [6], [46], [47]. The importance of such “stratification” in paternity insurance was notably proposed for the snow crab *Chionoecetes opilio* [48], [49] and the importance of position of stored sperm has been noted in fertilization success of isopods [50]. Moreover, our results show that 83% (5/6) of fathers present on the same host than female had the skewness of paternity in their favor (in clutch or because some alleles in spermatheca were not used). It is likely that this father was the last male that mated with the female. Therefore, anatomic and genetic data provide consistent arguments to suggest sperm precedence [39] for *D. primitivus*.

Future researches should test, for contrasted conditions, which factors particularly affect multiple paternity (e.g. predation pressure off hosts). Spermathecae should be further examined to characterize the degree of sperm stratification after short-term and long-term sperm storage, in order to estimate sperm precedence pattern. Moreover, a complementary experiment in aquarium (where all mating individuals are known) could also be done in order to quantify post-mating sexual selection and the sex difference in the opportunity for selection [2], [51].

## Supporting Information

Table S1 **Summary of the calculation of the mating skew in multipaternal clutches.** Mp  =  total number of fathers. SSq  =  sum of squares of the relative contributions. Me,p  =  effective number of fathers ( = 1/SSq). S  =  (Mp − Me,p)/(Mp -1)
(DOCX)

Table S2 **Estimate of the average number of offspring produced per male (and variance).** Total brood size of 203 was considered, because it is the average brood size calculated from female data.
(DOCX)
