# Supplementary material for: Genetic Evidence Confirms Polygamous Mating System in a Crustacean Parasite with Multiple Hosts
Source: PLoS One. 2014 Mar 7;9(3):e90680. doi: 10.1371/journal.pone.0090680 (PMC3946544; doi:10.1371/journal.pone.0090680)
Supplement: Table S1 — Summary of the calculation of the mating skew in multipaternal clutches. Mp = total number of fathers. SSq = sum of squares of the relative contributions. Me,p = effective number of fathers ( = 1/SSq). S = (Mp − Me,p)/(Mp -1) (DOCX) [file pone.0090680.s001.docx]

| **Mother's (clutch) ID** | **Relative contributions of each father** | **Mp** | **SSq** | **Me,p** | **S (skew)** |
| --- | --- | --- | --- | --- | --- |
| M12 | 0.978 : 0.022 | 2 | 0.96 | 1.04 | 0.96 |
| M16 | 0.636 : 0.364 | 2 | 0.54 | 1.86 | 0.14 |
| M49 | 0.838 : 0.162 | 2 | 0.73 | 1.37 | 0.63 |
| M64 | 0.833 : 0.056 : 0.028 : 0.028 : 0.028 : 0.028 | 6 | 0.70 | 1.43 | 0.91 |
| M69 | 0.886 : 0.086 : 0.029 | 3 | 0.79 | 1.26 | 0.87 |
| M70 | 0.844 : 0.133 : 0.022 | 3 | 0.73 | 1.37 | 0.82 |
| P3 | 0.976 : 0.024 | 2 | 0.95 | 1.05 | 0.95 |
| P5 | 0.867 : 0.089 : 0.044 | 3 | 0.76 | 1.31 | 0.84 |
| P11 | 0.951 : 0.049 | 2 | 0.91 | 1.10 | 0.90 |
| P59 | 0.857 : 0.143 | 2 | 0.75 | 1.32 | 0.68 |
| P69 | 0.867 : 0.067 : 0.044 : 0.022 | 4 | 0.76 | 1.32 | 0.89 |
| P70 | 0.636 : 0.205 : 0.159 | 3 | 0.47 | 2.12 | 0.44 |
