# Supplementary material for: Genetic Evidence Confirms Polygamous Mating System in a Crustacean Parasite with Multiple Hosts
Source: PLoS One. 2014 Mar 7;9(3):e90680. doi: 10.1371/journal.pone.0090680 (PMC3946544; doi:10.1371/journal.pone.0090680)
Supplement: Table S2 — Estimate of the average number of offspring produced per male (and variance). Total brood size of 203 was considered, because it is the average brood size calculated from female data. (DOCX) [file pone.0090680.s002.docx]

| **Male's  ID** | **Offspring tested  in clutch 1** | **Offspring fertilized in clutch 1** | **Total offspring  fertilized  in clutch 1** | **Offspring tested  in clutch 2** | **Offspring fertilized in clutch 2** | **Total offspring  fertilized  in clutch 2** | **Total offspring produced** |
| --- | --- | --- | --- | --- | --- | --- | --- |
| M15 | 46 | 45 | 198.59 |  |  |  | 198.59 |
| M24 | 31 | 31 | 203 |  |  |  | 203 |
| M31 | 39 | 39 | 203 |  |  |  | 203 |
| M38 | 47 | 47 | 203 |  |  |  | 203 |
| M63 | 36 | 30 | 169.17 |  |  |  | 169.17 |
| M71 | 45 | 6 | 27.07 |  |  |  | 27.07 |
| P27 | 37 | 37 | 203 |  |  |  | 203 |
| P45 | 37 | 37 | 203 |  |  |  | 203 |
| M18 | 36 | 2 | 11.28 |  |  |  | 11.28 |
| M84 | 36 | 1 | 5.64 | 35 | 31 | 179.80 | 185.44 |
| 1 | 46 | 1 | 4.41 |  |  |  | 4.41 |
| 2 | 44 | 28 | 129.18 | 35 | 3 | 17.40 | 146.58 |
| 3 | 44 | 16 | 73.82 |  |  |  | 73.82 |
| 4 | 37 | 31 | 170.08 | 44 | 7 | 32.30 | 202.38 |
| 5 | 37 | 6 | 32.92 |  |  |  | 32.92 |
| 6 | 36 | 1 | 5.64 | 42 | 41 | 198.17 | 203.81 |
| 7 | 36 | 1 | 5.64 | 35 | 1 | 5.80 | 11.44 |
| 8 | 36 | 1 | 5.64 | 42 | 1 | 4.83 | 10.47 |
| 9 | 45 | 38 | 171.42 |  |  |  | 171.42 |
| 10 | 45 | 1 | 4.51 |  |  |  | 4.51 |
| 11 | 45 | 39 | 175.93 |  |  |  | 175.93 |
| 12 | 45 | 2 | 9.02 |  |  |  | 9.02 |
| 13 | 45 | 4 | 18.04 | 45 | 39 | 175.93 | 193.98 |
| 14 | 41 | 39 | 193.10 |  |  |  | 193.10 |
| 15 | 41 | 2 | 9.90 |  |  |  | 9.90 |
| 16 | 43 | 43 | 203 | 35 | 30 | 174.00 | 377 |
| 17 | 35 | 5 | 29 |  |  |  | 29 |
| 18 | 45 | 3 | 13.53 |  |  |  | 13.53 |
| 19 | 45 | 2 | 9.02 |  |  |  | 9.02 |
| 20 | 45 | 1 | 4.51 |  |  |  | 4.51 |
| 21 | 44 | 28 | 129.18 |  |  |  | 129.18 |
| 22 | 44 | 9 | 41.52 |  |  |  | 41.52 |
| Mean (variance) |  |  |  |  |  |  | 114.19  (9677.30) |
